# The time and place of origin of South Caucasian languages: insights into past human societies, ecosystems and human population genetics

**DOI:** 10.1038/s41598-023-45500-w

**Published:** 2023-11-30

**Authors:** Alexander Gavashelishvili, Merab Chukhua, Kakhi Sakhltkhutsishvili, Dilek Koptekin, Mehmet Somel

**Affiliations:** 1 Center of Biodiversity Studies, Institute of Ecology, Ilia State University, Cholokashvili Str. 5, 0162 Tbilisi, Georgia; 2 Head of the Institute of Caucasiology, Faculty of Humanities, Ivane Javakhishvili Tbilisi State University, Ilia Chavchavadze Str. 1, 0162 Tbilisi, Georgia; 3 Georgian DNA Project, Family Tree DNA, Ilia State University, Cholokashvili Str. 5, 0162 Tbilisi, Georgia; 4 Department of Biological Sciences, Middle East Technical University, 06800 Ankara, Turkey

**Keywords:** Computational biology and bioinformatics, Ecology, Genetics, Ecology

## Abstract

This study re-examines the linguistic phylogeny of the South Caucasian linguistic family (aka the Kartvelian linguistic family) and attempts to identify its Urheimat. We apply Bayesian phylogenetics to infer a dated phylogeny of the South Caucasian languages. We infer the Urheimat and the reasons for the split of the Kartvelian languages by taking into consideration (1) the past distribution ranges of wildlife elements whose names can be traced back to proto-Kartvelian roots, (2) the distribution ranges of past cultures and (3) the genetic variations of past and extant human populations. Our best-fit Bayesian phylogenetic model is in agreement with the widely accepted topology suggested by previous studies. However, in contrast to these studies, our model suggests earlier mean split dates, according to which the divergence between Svan and Karto-Zan occurred in the early Copper Age, while Georgian and Zan diverged in the early Iron Age. The split of Zan into Megrelian and Laz is widely attributed to the spread of Georgian and/or Georgian speakers in the seventh-eighth centuries CE. Our analyses place the Kartvelian Urheimat in an area that largely intersects the Colchis glacial refugium in the South Caucasus. The divergence of Kartvelian languages is strongly associated with differences in the rate of technological expansions in relation to landscape heterogeneity, as well as the emergence of state-run communities. Neolithic societies could not colonize dense forests, whereas Copper Age societies made limited progress in this regard, but not to the same degree of success achieved by Bronze and Iron Age societies. The paper also discusses the importance of glacial refugia in laying the foundation for linguistic families and where Indo-European languages might have originated.

## Introduction

**Figure 1:**
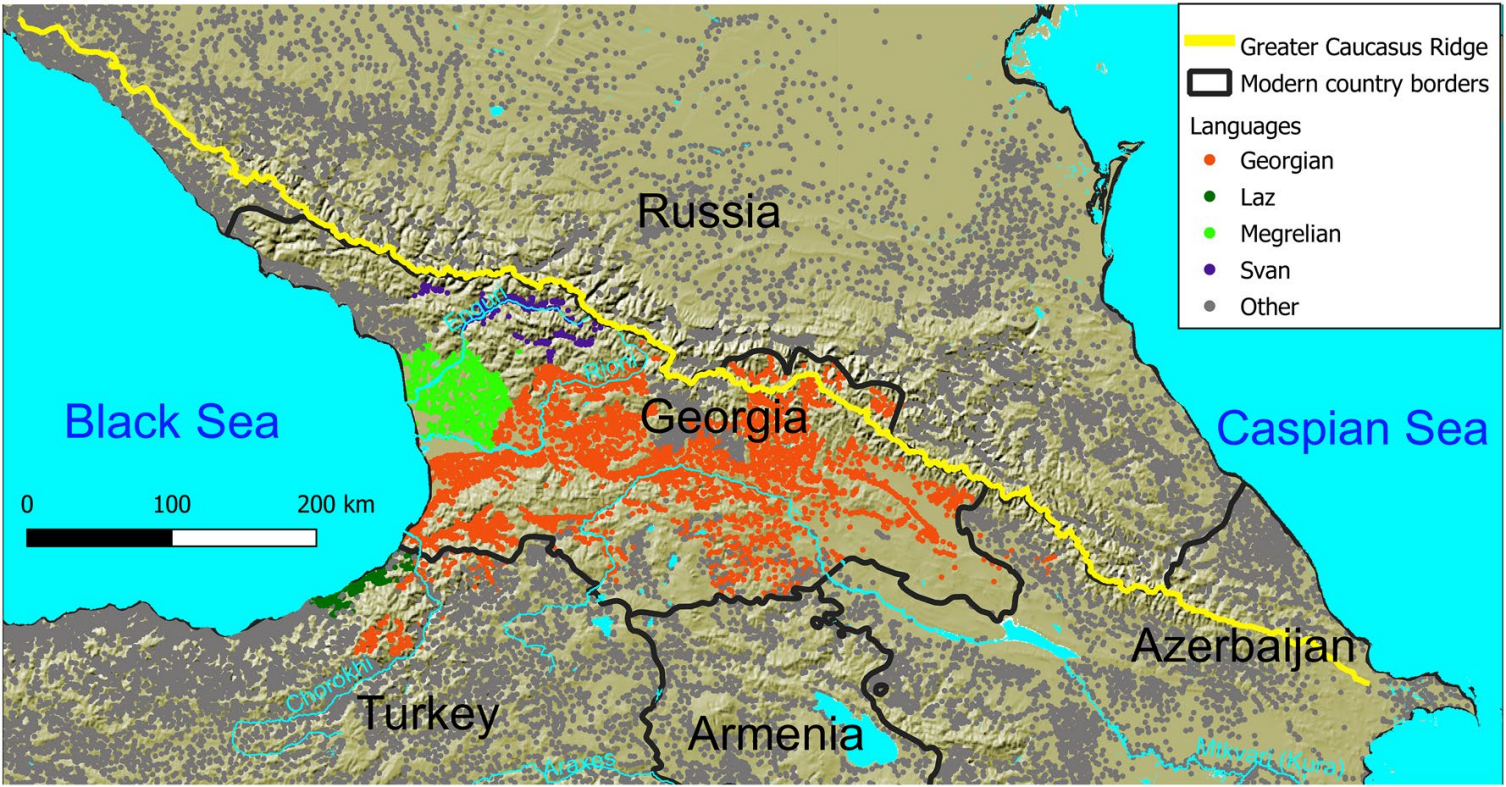
The distribution of South Caucasian languages in the twentieth and twenty-first centuries. Points depict human settlements whose dominant languages are identified by colors. The main ridge of the Greater Caucasus divides the Caucasus region into the South Caucasus and the North Caucasus. The map is generated using QGIS Desktop 3.22.7-Białowieża (https://qgis.org).

Based on the reconstructed proto-words of several Eurasian language families, proto-Kartvelian is suggested to have emerged over 12,500 BP (Before Present standing for years before 1 January 1950), predating proto-Indo-European, proto-Uralic, proto-Altaic, proto-Inuit-Yupik and proto-Chukchi-Kamchatkan^1^. Currently, the Kartvelian language family (aka the South Caucasian language family) consists of only four extant languages: Georgian, Svan, Megrelian and Laz, with Georgian being the most widely spoken among them. The majority of Kartvelian speakers live in the country of Georgia and northeastern Turkey (Fig. 1). The Megrelian and Laz languages constitute a branch of the South Caucasian languages, which is termed Zan. Most scholars accept the South Caucasian family tree, in which Svan is sister to the clade of the remaining three languages. The application of lexicostatistics and glottochronology for the classification and timing of South Caucasian languages dates the split of the Proto-Kartvelian into Svan and Proto-Georgian-Zan (aka Proto-Karto-Zan) to 3950–4150 BP^2–4^, 4750 BP^5^, 4400 BP^6^, 4190 BP^7^ and the further division into Georgian and Zan to 2550–2650 BP^3,4^, 2750 BP^5^, 2000 BP^6^, 1850 BP^7^. Sergei Starostin's unpublished model pushes dates further back to 4990 BP for the split between Svan and Proto-Georgian-Zan, 2730 BP for the split between Georgian and Zan, and 1330 BP for the split between Laz and Megrelian^7^. Notably, these studies do not provide measures of uncertainty or validation against known facts that are not used as prior information in model fitting. The split of Zan into Megrelian and Laz was caused by the massive spread of Georgian and/or Georgian speakers from the watershed of Mtkvari (Kura) River to those of Chorokhi and Rioni Rivers, which started in the mid-seventh century CE and peaked in the eighth century CE—that is, 1250—1150 years BP^8,9^. Nowadays Bayesian phylogenetic inference is preferred over lexicostatistics^10–13^. In contrast to lexicostatistics, Bayesian phylogenetic methods (1) consider the distinction between shared retentions and shared innovations, (2) account for rate variation between parts of the data, between lineages, and over time and (3) are robust to the effects of borrowing as they quantify the uncertainty in their estimates of parameters and tree topologies^14^. To our knowledge Bayesian phylogenetic methods have never been applied to infer a dated phylogeny of the South Caucasian languages before.

On the basis of analyzing archaic lexical and toponymic data, Gamkrelidze and Ivanov^8^ hypothesized that Proto-Kartvelian emerged in the western and central parts of the Lesser Caucasus in the 4th and 3rd millennia BCE; the first wave of migrants from this homeland moved to the Black Sea basin in the South Caucasus (i.e. what is now western Georgia) in the 3rd millennium BCE and their language evolved into Svan; the next wave of migrations in the 2nd millennium BCE from the Kartvelian homeland to the Black Sea basin led to the formation of Zan that gradually displaced Svan to the north; dialects of the people remaining in the Kartvelian homeland evolved into Georgian, speakers of which spread across the South Caucasus in the historical times, largely displacing Zan and splitting it into Laz and Megrelian, as well as displacing Svan and Northeast Caucasian languages in some areas. According to historical records, from the eighth century BCE through the first century CE Svan was spoken in most of what is now western Georgia (aka Colchis in the past), Zan was spoken in the Chorokhi (Çoruh) River basin and much of what is now Turkey’s Black Sea Region, while Georgian was spoken in the Caspian Sea basin, mostly in what is now eastern Georgia^9,15^. According to some theories, there is a probable association between the South Caucasian languages and a pre-Greek substrate^16^. The proposed simple method, that only considers linguistic and geographical distances between languages to locate homelands of linguistic families, provides a very rough picture of point-based homelands of the Caucasian linguistic families^17^.

The inferred dates for the split between Svan and Proto-Georgian-Zan, the suggested location of the Kartvelian homeland and the proposed scenario of Kartvelian migrations fail to align with the timing of the emergence of vocabulary related to crop cultivation, herding and metallurgy in the Kartvelian languages. Cattle-breeding vocabulary and terms for wine are common to Svan and the other Kartvelian languages, whereas terms for crop cultivation, sheep-breeding and metallurgy, which are common to the other Kartvelian languages, are absent in Svan^4^, (Supplementary Data 1). Both archaeological and genetic evidence suggest that crop cultivation and herding began approximately 12,000 BP in the Fertile Crescent and gradually spread across the Near East and the Caucasus by c. 8,000 BP. The domestication of cattle, specifically extinct Eurasian aurochs (*Bos primigenius*), occurred ~ 10,500 BP and subsequently spread throughout the Near East by the end of the Neolithic period^18,19^. The wild ancestor of domestic sheep is thought to have been the Asian Mouflon (*Ovis orientalis*) whose management and domestication began c.12,000–10,000 BP^20^. Crop cultivation and processing (e.g. emmer and einkorn wheat, barley, pea, lentil, legume) began to spread 12,000–11,000 BP through the movements of Neolithic Anatolian farmers^21,22^. Both archaeological and genetic evidence also suggest that the earliest production of wine took place in or near the South Caucasus c. 8000 BP^23^. Thus, the vocabulary of wine, cattle-breeding and metallurgy places the split between Svan and Proto-Georgian-Zan in the Chalcolithic period (i.e. 8000–5000 BP) in or near the South Caucasus. Consequently, this scenario proposes that the split between Svan and Proto-Georgian-Zan took place earlier than the dates inferred through the application of lexicostatistics and glottochronology (as mentioned above). However, the vocabulary of crop cultivation and sheep-keeping does not agree with this, implying that the split between Svan and Proto-Georgian-Zan occurred either before the Neolithic period or before these practices reached the Kartvelian homeland within the general area of the Near East and the Caucasus. This is unrealistic because these practices began to spread from the Fertile Crescent slightly earlier than, or about the same time as, cattle-breeding did. The plausible scenario for this mismatch is that crop cultivation and sheep-breeding took much longer time to penetrate the Kartvelian homeland than cattle-breeding, and by the time crop cultivation and sheep-breeding did so, the linguistic split had already occurred. This difference in the rate of spread between farming practices could be explained by landscape heterogeneity, with some landscape types favoring cattle-breeding and some other landscape types favoring crop-cultivation and sheep-breeding. If this is true, then at least some part of the Kartvelian homeland initially was unsuitable for crop-cultivation and sheep-breeding, and the period between the split and the introduction of crop cultivation, sheep breeding and metallurgy was long enough to solidify the linguistic differences. Additionally, we assume that wildlife elements, whose current names can be traced back to proto-Kartvelian names without semantic changes, co-occurred in the Kartvelian homeland.

To analyze the validity of these assumptions, we attempt (1) to time the origin of Kartvelian languages by analyzing their phylogeny using Bayesian phylogenetic inference, (2) to locate the Kartvelian homeland by inferring the past geographic ranges of animals and plants whose names are reconstructible to proto-Kartvelian names, (3) to locate the Kartvelian homeland and infer the reasons for the split of the Kartvelian by analyzing the association of archaeological cultures with biomes, and (4) align past migrations as plausible vectors for linguistic changes with linguistic phylogeny by analyzing the genetic variations of past and extant human populations.

In addition to being the first to apply Bayesian phylogenetic inference to the Kartvelian language family, this paper demonstrates an innovative approach that combines linguistics, archaeology, landscape ecology, human population genetics and biodiversity studies to validate linguistic phylogenies and locate language homelands. Our approach provides an opportunity to re-examine and improve the existing models of such complex linguistic groups as Northwest and Northeast Caucasian languages. There is linguistic evidence that points either to possible structural relationship or to prolonged contacts between Kartvelian and Indo-European languages in the South Caucasus^4,8^. This is supported by recently discovered genetic evidence of a ghost population in or near the South Caucasus, which acted as the link connecting the Proto-Indo-European-speaking Yamnaya with the speakers of Anatolian languages^24^. In this context our findings will help reduce the search area for the homeland of Indo-European languages and provide more clarity about the nature of ties between Kartvelian and Indo-European languages.

## Materials and methods

### Language data

We compared basic vocabulary of 254 meaning concepts (i.e. meaning classes) across the Kartvelian languages: Georgian, Old Georgian, Megrelian, Laz and Svan. Of these languages all but Old Georgian are spoken today. According to historical records Old Georgian existed until ~ 900 years ago^9,25–31^. These meaning concepts were extracted from the merger of the Leipzig-Jakarta 200 list^32^ and the Jena 200 list^33^. We used most generic terms for the semantic specification of basic vocabulary concepts as defined in Savelyev and Robbeets^34^. Meaning concepts for ‘go (v.)’, ‘come (v.)’ and ‘walk (v.)’ were combined because in the Kartvelian languages these verbs derive from the same roots for general movement, whose specific meanings vary with prefixes. Meaning concepts for ‘3SG pronoun’ and ‘That’ were also combined because they are the same in the Kartvelian languages. So, we ended up with 251 meaning classes for our analyses. More than one word was used to represent each meaning concept in a given language (i.e. more than one cognate set). So, we compiled synonymous cognate sets for each meaning concept. Borrowings were eliminated using comparative etymological dictionaries^4,35,36^, and knowledge of sound laws that reliably allows the detection of borrowings^2,37–39^. Additional dictionaries^40–44^ were scrutinized to minimize uncertainties in concept and cognate definitions. This yielded 736 cognate sets covering 251 basic vocabulary meanings across the Kartvelian languages. Each item of the cognate set was coded as present (1), absent (0) or uncertain (?) for all languages in the dataset (Supplementary Data 2).

### Phylogenetic linguistic inference

We inferred posterior distributions of phylogenetic trees using a Bayesian Markov-chain Monte Carlo (MCMC) approach applied to the binary data through BEAST 2 with the Babel package^45^. We performed model fitting and optimization following Hoffmann et al. ^14^. We performed ascertainment correction for each meaning concept to compensate for latent cognates (i.e. those not observed in any of the languages in our sample)^46,47^. More specifically, the ascertainment correction was done by adding a single all-zero cognate set to each meaning concept. We tested three different models of cognate evolution (i.e. models of cognate gain and loss) that have previously been applied to cognate data: binary continuous time Markov chain (CTMC), binary covarion (BC) and stochastic Dollo (SDollo). CTMC allows cognates to be gained and lost at the same rate, being analogous to the HKY simple nucleotide substitution model^48^. BC allows each cognate set to remain relatively stable over time but occasionally switch into a faster rate of change on different branches, which implies that certain words may change faster across parts of the tree^49^. SDollo allows cognates to appear once on a tree but get lost multiple times^12^. We used the Fossilized Birth Death tree prior^50^ appropriate for data in which some languages might not survive to the present. To calibrate the clock, we applied 900 years BP to Old Georgian, which is the last seen date identified by linguists^9,25–31^. A normal time constraint of 1200 −/ + 10 years BP was put on the Zan internal node—i.e. a prior for the split time between Laz and Megrelian (see the introduction). No time constraints were put on other internal nodes in the Kartvelian tree because reliable historical clues were not available to make any proper assumptions. Dated trees were then inferred under the strict clock model and the uncorrelated lognormal relaxed clock. The strict clock model assumes that every branch in the tree evolves at the same evolutionary rate, while the uncorrelated lognormal relaxed clock allows for variations in rates across branches. We tested evolution rate variations across sites that implied that each site (= cognate set) in a meaning concept had its rate shared with other sites in the same concept, but each concept had its own rate, thus allowing the rate variation between meaning concepts. CTMC and SDollo models were run both with and without gamma-distributed rate heterogeneity (four categories) across cognates. So, our procedure produced 10 models. To select the best model for our data, we estimated the marginal likelihood (ML) using the nested sampling^51^ with 20 particles. From these estimates, we calculated Bayes factors (BF) to determine which model best fitted our data. A log-transformed BF of at least 5, where BF is the ratio of marginal likelihoods of model 1 to model 2, indicates very strong support for model 1 over model 2^52^.

We ran 200 million generations of MCMC chains, sampling trees every 5000 generations to minimize autocorrelation, resulting in a sample of 40,000 trees. A burn-in of 10% of the iterations was removed leaving a posterior sample of 36,000 trees. Autocorrelation, convergence and the estimated sample size (ESS) were checked using Tracer v. 1.6^53^. ESS was well over 200 for the posterior and all the other important parameters, including the prior, the likelihood, and the tree height. The maximum clade credibility (MCC) tree was derived using TreeAnnotator v2.6.0^54^.

### Kartvelian homeland

The Kartvelian homeland must have been the area where before or at the time of the split of Proto-Kartvelian there was the overlap of the geographic ranges (i.e. co-occurrence) of animals and plants (hereafter the taxa), whose names are reconstructible to proto-Kartvelian names (i.e. to their common roots). To identify these ranges and their overlaps, we derived taxon habitat suitability models in relation to current climate, and projected these taxon-climate models onto rasterized climatic predictors reconstructed for the time span between 1500 and 15,000 BP (hereafter the study time span) across the Near East, the Caucasus, the Balkans, the Pontic-Caspian steppe and part of the Central Asia (hereafter the study area). The study encompassed a temporal range extending from the Mesolithic/Epipalaeolithic period to the Iron Age.

For climatic predictors we used two variables: (1) mean annual temperature as a simple comparative measure of warmth and the length of the growing season and (2) annual precipitation as a measure of water availability. We used these two climate variables because they sufficiently explain global variation in vegetation communities^55,56^ that in turn account for animal communities. As climatic predictors for the development of taxon-climate habitat suitability models, we used climatic raster layers downloaded from the CHELSA climatology data, a set of global climate layers with a spatial resolution of 1 km^2^, which provides various parameters of temperature and precipitation at a global scale for various time periods, ranging from the Last Glacial Maximum, to the present, to several future scenarios^57^.

Taxon occurrence points, with coordinate uncertainty less than 50 m, were obtained from Georgia's 2019–2021 national forest inventory data (source: the National Forest Agency of Georgia), the Global Biodiversity Information Facility data (GBIF.org, download 25 February 2023) and our fieldwork data. Taxon occurrence points in urban areas, museums, botanical gardens, parks, herbaria as well as duplicated ones were removed from these data sets to minimize sampling bias and human influence on the taxon distribution.

For our study, we selected 22 taxa, (1) whose names were reconstructible to their common roots, (2) whose distributions could be modeled in relation to climate, and (3) which did not occur widely across the study area and the study time span, and hence could be good for identifying linguistic homeland (Table 1). The selected taxa represented taxonomic groups with different life cycles, physiologies, reproduction and dispersal potential.

**Table 1:** Concepts of taxa used to identify the Kartvelian homeland (see Supplementary Data 1 for details). The list of Laz lacks some key terms because they have been replaced with Turkish terms since the Ottoman era before being recorded.

Concept	Georgian	Svan	Megrelian	Laz
Chamois (*Rupicapra spp.*)	arčvi	hersḳn	erckemi	
Yellow Azalea (*Rhododendron luteum*)		hadra	odi	odi
Goat willow (*Salix caprea*)	poxvi	pixvra	purxi	
	bagvi	bægwra	buguʒiri	
Cherry laurel (*Prunus laurocerasus*)	c̣q̇avi, mc̣q̇avi	c̣q̇aw, c̣q̇ew	c̣q̇ili, c̣q̇i, c̣q̇ivi, c̣q̇ovi, c̣q̇oi	mc̣ḳo, mc̣ḳoli, c̣u
Dwarf elder (*Sambucus ebulus*)	anc̣li, anc̣liḳa	ganč̣w, gænč̣w	inč̣iria, inč̣ilia, inč̣iri	inč̣ira, inč̣iri
Chestnut (*Castanea sativa*)	c̣abli	c̣ebeld	č̣uburi	č̣uburi, č̣ubui
Aspen (*Populus tremula*)	verxvi	werxw, werxwla, jerxwla	vexi	
Linden (*Tilia spp.*)	cacxvi, cacx	zæsxw, zesxa	ducxu	ducxu
Caucasian rhododendron (*Rhododendron caucasicum*)	zisxa	zišxora	žiške	
Medlar (*Mespilus germanica*)	zɣmarṭli, žɣmarṭli, zimarṭli, simarṭli, sxmarṭlin, simarṭl	zunṭi, zunki, žuunṭu	ckumunṭuri, cxumunṭuri	ckirmuṭuri
Colchic butcher's-broom (*Ruscus colchicus*)	ʒmerxli, ǯmerxli, ǯilmexi	zəmex	zormexi	zermexi, zurmexi, zurmex
Sycamore (*Acer pseudoplatanus*)	teḳera	teḳra, teḳər	taḳveri	taḳveri
Ash (*Fraxinus excelsior*)	ipani, ipeni, ipni, ipna	ip, ipar, ipnaj	liponi	
		læǯra, lenǯ	laǯi, lanǯi	
Spruce (*Picea orientalis*)	naʒvi	nezwra, nenz	nuzu	nuzu
Birch (*Betula spp.*)	ʒaxveli, ʒaqueli	žaqwer, žæqwra, ǯaq̇varla		
Yew (*Taxus baccata*)	utxovari, urtxmela	urtxa, utxa	urtxeli	urtxeli
Maple (*Acer spp.*)	meḳencxali, neḳerčxali, neḳerčxali, naḳerčxali, ḳemerčxali, ḳimerčxali, ḳenerčxali, meḳerčxali, leḳenčxali, leḳončxali, meḳenčxali, neḳuerčxal, leḳmaḳenčxa	biḳenčxal, biḳinčxal	laḳinčxa	
Pontic rhododendron (*Rhododendron ponticum*)	šiari	šgeri, šgoori	pšḳeri, škeri	mškeri
Hazel (*Corylus spp.*)	txili	šdix, šṭix, šdixənd, šdəxənd	txiri	mtxiri, ntxiri
Hornbeam (*Carpinus spp.*)	rcxila, cxilai, cxumi, cxomi, cxemla, rcxemla, krcxila	cxwim, cxəmra, cxum	cxomi, cxemuri, cximuri	cxomi, cxemuri, cximuri, mcxubri
Elm (*Ulmus spp.*)	cvela	cæjra, caajra	cə	
Beech (*Fagus spp.*)	c̣ipeli	c̣ip, c̣ipra	c̣ipuri	c̣ipuri, eipra

To model the distribution of all the taxa throughout the study area and the study time span, we used maximum entropy (MaxEnt) modeling^58^. MaxEnt models the probability of species distribution or the species habitat suitability by contrasting distributions of environmental predictors at taxon occurrence points with distributions of these predictors within the available landscape (i.e. at random background points), while using regularization parameters to prevent overfitting^59,60^. MaxEnt generally outperforms concurrent algorithms and works better with a broad spectrum of datasets^61^. We used MaxEnt v. 3.4.1 with a maximum of 2500 iterations, quadratic and hinge features only, and default settings for convergence thresholds and regularization^60,62^. We generated a random sample of 10,000 background points across the study area. To avoid the repeated sampling of rasterized environmental predictors, occurrence points (Supplementary Data 3) and background points (Supplementary Data 4) were selected such that there was one point per pixel of the aligned raster layers of environmental predictors. To map the spatial overlap of the distributions of the taxa, we first converted the resulting habitat suitability map of each taxon into a binary presence/absence map using the maximum training sensitivity plus specificity threshold^63^ and then multiplied these binary maps.

Additionally, to locate the Kartvelian homeland and infer the reasons for the split of the Kartvelian, we analyzed the association of archaeological cultures with biomes across the study area and the study time span. Data on 1229 dated archaeological sites (Supplementary Data 5) as well as data on 1134 dated sites of fossil pollen composition and land cover types (Supplementary Data 6) were obtained from various sources. We grouped the sites of archaeological societies into five cultures: Hunter-gatherers (HG), Neolithic (N), Copper Age (CA), Bronze Age (BA) and Iron Age (IA). Fossil pollen data and land cover sites were grouped into 8 biomes. Three of these biomes included the ones with < 5% arboreal pollen—i.e., Desert, Steppe, Tundra and Glacier. The rest (i.e.5 biomes) had >  = 5% arboreal pollen (AP): biome with 5–25% AP, biome with 25–50% AP, biome with 50–75% AP and biome with > 75% AP. The biomized sites were linked spatially and temporally to the two climatic predictors from the CHELSA climatology data^57^. Multinomial logistic regression (MLR) was used to evaluate the relationships between the biomized sites and the climatic predictors using the nnet package^64^ in R version 4.2.2^65^. We used multinomial logistic regression rather than discriminant analysis or random forests because the former is unable to find relationships without making assumptions and the latter is a "black box" method, and hence researchers have very little control of what the algorithm does^66^. The derived biome-climate model was mapped by projecting it onto a time-series of CHELSEA-reconstructed climate raster maps. The past societies were plotted in relation to inferred biomes over the study time span, using the "ggplot2" package^67^ in R version 4.2.2. The inferred society-biome associations were mapped to visualize our hypotheses. All maps in this study were generated using QGIS Desktop 3.22.7-Białowieża (https://qgis.org).

### Population genetics

To align past migrations as plausible vectors for linguistic and technological changes with linguistic phylogeny, we analyzed the genetic variations of past and extant human populations using principal component analysis (PCA) of humans genotyped for genome-wide autosomal SNPs (single nucleotide polymorphisms). PCA is used to detect the presence of population structure, identify differences in ancestry among populations and samples, regardless of the history or processes underlying the structure, and provide evidence of migration events^68^. We used the "smartpca" program version 18,140 of "EIGENSOFT" version 7.2.0^69^ with "lsqproject:YES, numoutlieriter: 0" parameters to construct the principal components of modern West Eurasian populations from Human Origins SNP Array dataset^70^. Ancient individuals from Koptekin et al.^70^ and linguistically explicit samples from Gavashelishvili et al.^71^ were projected onto the first two principal components of present-day genetic variance (Supplementary Data 7). To maximize the representativeness of the genetic signature of each language-speaking population, the linguistically explicit samples were collected from locals with no ancestors from outside of the respective language-speaking population at least over the last five generations.

## Results

**Figure 2:**
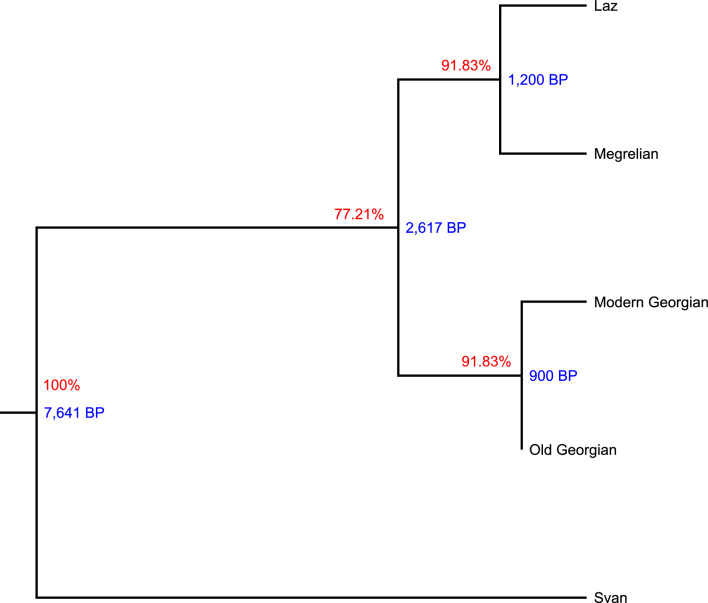
Maximum Clade Credibility tree of Kartvelian languages, inferred using the Bayesian phylogenetic model driven by binary covarion cognate evolution and a relaxed clock. Posterior probabilities (%) and average split ages (in years BP) of internal nodes are shown.

According to all of our Bayesian phylogenetic linguistic models, Svan was found to be sister to the clade of the other three languages. All models inferred the mean root age of > 4000 BP. Strict clock models inferred greater ages than relaxed clock models (Supplementary Fig. S1). The binary covarion cognate evolution model with a relaxed clock had the best fit for the dataset (Table 2). The model yielded high posterior probabilities for the root and all nodes (Fig. 2). In terms of chronology, our best-fit model estimated the mean root age to be 7641 BP (95% highest posterior density (HPD): 18,626–1169 BP) for the split between Svan and Karto-Zan. The mean age for the split between Georgian and Zan was estimated at 2617 BP (95% HPD: 4323–1178 BP). The mean age for the split of Zan into Megrelian and Laz was estimated at 1200 BP (95% HPD: 1219–1180 BP). Increase in sigma on the Zan prior to values of 50, 100, 200 only increased uncertainty and did not affect the mean root and node ages sufficiently to question our conclusions made in relation to other components of our study.

**Table 2:** Comparison of the fit of different linguistic models by estimating the marginal likelihoods using nested sampling in Bayesian phylogenetic linguistics.

Model of cognate evolution	Site model Gamma rate categories	Clock model	Marginal likelihood	SD	Bayes factor	log BF
Binary Covarion	1	Relaxed clock Log Normal	− 2034.34	1.92	1	0.00
Binary Covarion	1	Strict clock	− 2062.58	1.96	1.84E + 12	28.24
binary CTMC	1	Relaxed clock Log Normal	− 2070.56	2.46	5.37E + 15	36.22
binary CTMC	4	Relaxed clock Log Normal	− 2071.89	2.24	2.04E + 16	37.56
binary CTMC	4	Strict clock	− 2082.68	2.29	9.89E + 20	48.34
binary CTMC	1	Strict clock	− 2091.45	2.53	6.39E + 24	57.12
SDollo	1	Relaxed clock Log Normal	− 2532.24	1.43	1.72E + 216	497.90
SDollo	4	Relaxed clock Log Normal	− 2545.32	1.60	8.30E + 221	510.99
SDollo	1	Strict clock	− 2546.02	1.27	1.66E + 222	511.68
SDollo	4	Strict clock	− 2561.48	1.61	8.62E + 228	527.14

**Figure 3:**
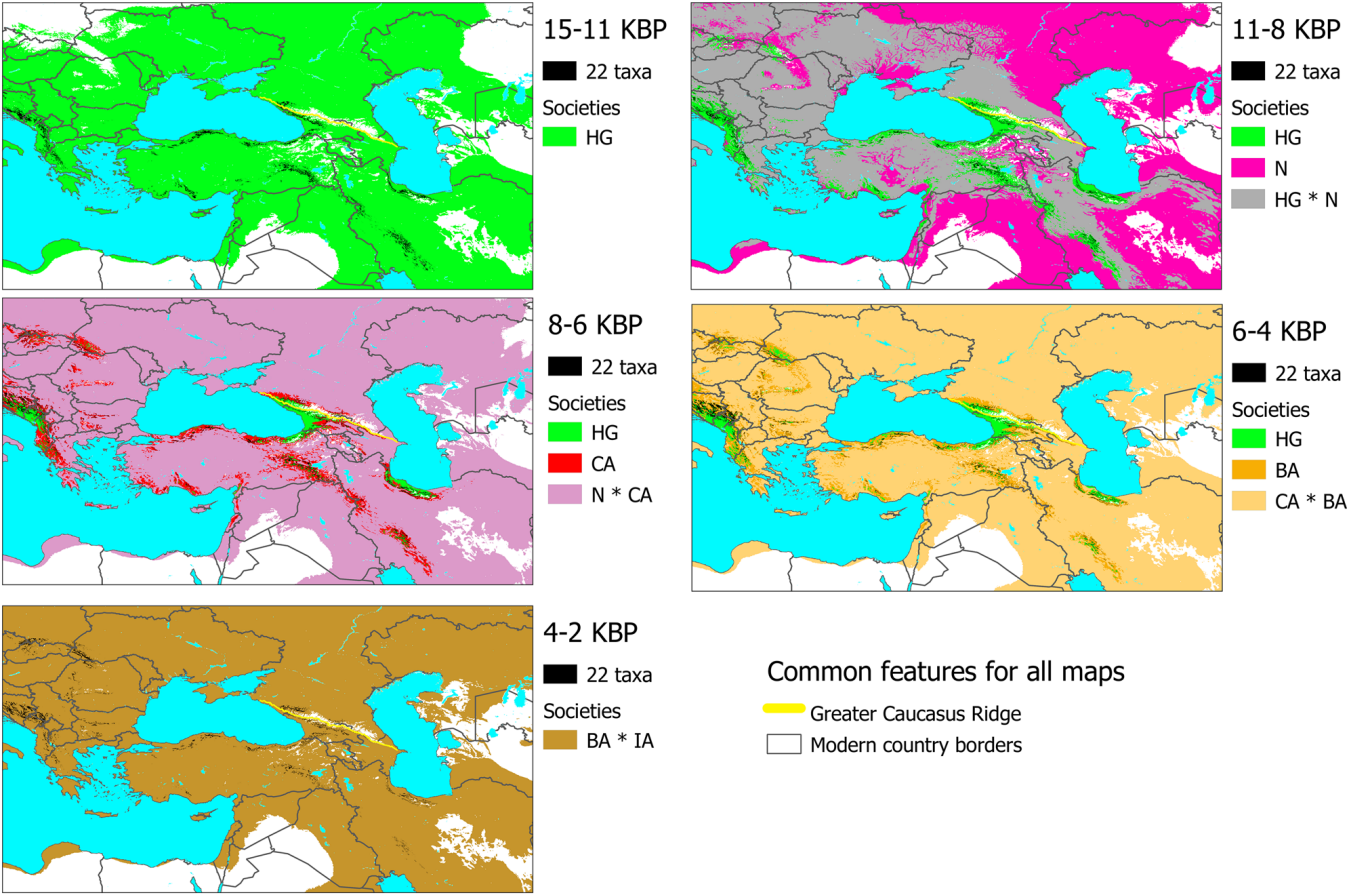
Co-occurrence of wildlife elements, whose names are reconstructible to proto-Kartvelian names, and the distribution of the past human societies. The taxa co-occurrence is inferred by mapping each of the MaxEnt taxon-climate habitat suitability models, and subsequently binarizing and multiplying these maps. The co-occurrence areas were identified across CHELSA climatology time series, and then combined for each of the 5 time periods. The distribution of the past human societies is inferred from their associations with biomes (see Table 3 for details). The past societies are as follows: HG: Hunter-gatherers, N: Neolithic societies, CA: Copper Age societies, BA: Bronze Age societies, IA: Iron Age societies. The past human societies are mapped from biomes that are modeled across CHELSA climatology time series, and then aggregated for each of the 5 time periods using the mode value. The acronym of BP, denoting “Before Present”, stands for years before 1 January 1950. The maps are generated using QGIS Desktop 3.22.7-Białowieża (https://qgis.org).

The projection of the MaxEnt taxon-climate models onto a time series of climatic predictors generated the habitat suitability maps of the 22 taxa across the study area and the study time span. The subsequent binarization and multiplication of these maps identified the areas where wildlife elements, whose names are reconstructible to proto-Kartvelian names, co-occurred (Fig. 3; Supplementary Fig. S2). Notably, these areas were primarily concentrated in the mountainous regions of the Caucasus, Pontic Alps, Alborz, Zagros, Taurus, Mount Lebanon, Dinaric Alps, Pindus, Stara Planina, Rhodopes, and the Carpathians. However, throughout the entire study time span, they were consistently present in the Western Caucasus, Pontic Alps, Alborz, Zagros, Taurus, Mount Lebanon, Dinaric Alps, and Pindus. These areas appeared in the Eastern Caucasus, Stara Planina, Rhodopes and the Carpathians after 8000 BP.

**Figure 4:**
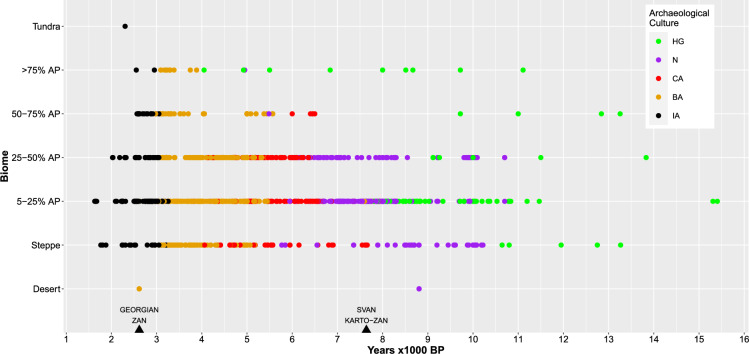
The distribution of the past human societies in relation to biomes and time across the Near East, the Caucasus, the Balkans, the Pontic-Caspian steppe and part of Central Asia. The past societies are as follows: HG: Hunter-gatherers, N: Neolithic societies, CA: Copper Age societies, BA: Bronze Age societies, IA: Iron Age societies. Biomes are inferred from fossil pollen sites and climate variables (see details in the text). AP stands for arboreal pollen, while BP, denoting “Before Present”, stands for years before 1 January 1950. The mean split ages between Svan and Karto-Zan and between Georgian and Zan, inferred through the best-fit model of the Bayesian phylogenetic linguistic method, are plotted on the x-axis.

The MLR biome-climate model correctly classified 76% of all sites, with a Kappa value of 0.717 (*P* < 0.0001). All biomes were correctly classified between 37.92 and 93.70% of all sites (Supplementary Table S1). The model worked best with 5–25% AP and worst with Steppe that is in line with other similar studies^72^. The correctly classified frequency of each category was the highest. The analysis of the association between archaeological cultures and the inferred biomes revealed substantial differences in the rate of technological expansions in relation to biomes (Fig. 4). From 15,500 to 11,000 BP, there were only hunter-gatherers and they occurred in steppe and all forests. From 11,000 to 8000 BP, hunter-gatherers were mainly present in all forests, and those in steppe were soon replaced by Neolithic societies. The distribution of Neolithic societies covered steppe and forests with less than 50% arboreal pollen. Between 8000 and 6000 BP, hunter-gatherers were only present in forests with greater than 75% arboreal pollen. Neolithic societies remained in the same biomes as before and overlapped with Copper Age societies, which were also present in forests with less than 75% arboreal pollen. From 6000 to 4000 BP, hunter-gatherers continued to occupy forests with greater than 75% arboreal pollen, and the remaining Neolithic societies were soon replaced by Copper Age societies. Copper Age and Bronze Age societies occupied steppe and forests with less than 75% arboreal pollen. Finally, from 4000 to 1500 BP, hunter-gatherers and Copper Age societies disappeared, while Bronze Age and Iron Age societies occupied almost all biomes. We used these associations (Table 3) in order to map the geographic distribution of these societies, more precisely the habitat suitability of these societies (Fig. 3; Supplementary Fig. S2).

**Table 3:** Associations between the past human societies and biomes across the Near East, the Caucasus, the Balkans, the Pontic-Caspian steppe and part of Central Asia over the study time span. The acronym of BP (i.e. Before Present) stands for years before 1 January 1950. Biomes are inferred from fossil pollen sites and climate variables. AP stands for arboreal pollen (see details in the text).

Time BP	Hunter-gatherers	Neolithic societies	Copper age societies	Bronze age societies	Iron age societies
15,000–11,000	Steppe 5–25% AP 25–50% AP 50–75% AP > 75% AP	None	None	None	None
11,000–8000	5–25% AP 25–50% AP 50–75% AP > 75% AP	Steppe 5–25% AP 25–50% AP	None	None	None
8000–6000	> 75% AP	Steppe 5–25% AP 25–50% AP	Steppe 5–25% AP 25–50% AP 50–75% AP	None	None
6000–4000	> 75% AP	None	Steppe 5–25% AP 25–50% A	Steppe 5–25% AP 25–50% AP 50–75% AP	None
4000–2000	None	None	None	Steppe 5–25% AP 25–50% AP 50–75% AP > 75% AP	Steppe 5–25% AP 25–50% AP 50–75% AP > 75% AP

**Figure 5:**
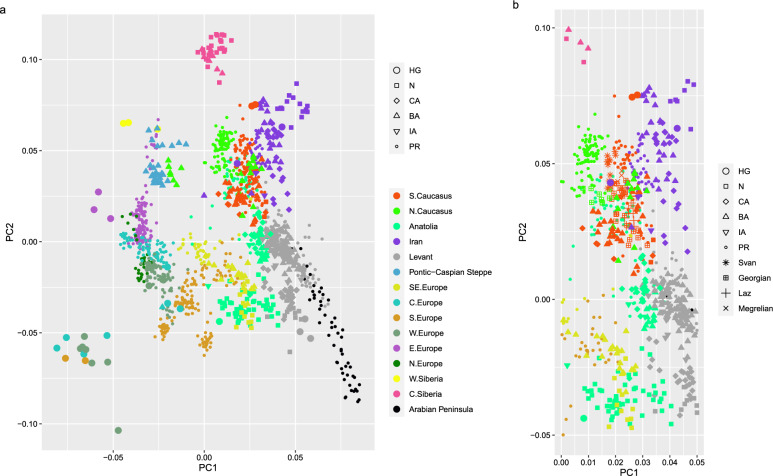
Principal component analysis (PCA): (**a**) all data points and (**b**) a close-up of speakers of modern Kartvelian languages. The plot shows the first two principal components calculated using genomes of 969 individuals from modern West Eurasian populations^70^, onto which a total of 478 ancient individuals from the Mesolithic/Epipalaeolithic period to the Iron Age^70^ and 85 linguistically explicit modern individuals^71^ are projected. Data point colors differentiate geographic regions, while shapes identify archaeological periods (HG: Hunter-gatherer, N: Neolithic, CA: Copper Age, BA: Bronze Age, IA: Iron Age, PR: Present-day and speakers of 4 modern Kartvelian languages. PC1 correlates with the north–south differentiation, whereas PC2 correlates with the east–west differentiation across different periods. This plot can be explored more easily using the supplementary interactive PCA plot.

Principal component analysis (PCA) of genome-wide SNP genotypes indicated that PC1 correlated with the north–south differentiation, whereas PC2 correlated with the east–west differentiation across different periods. Most of the modern Kartvelian speakers fell within the range of genetic variation of modern South Caucasians, being surrounded by modern North Caucasians, Iranians, Anatolians and Levantines (Fig. 5, Supplementary Fig. S3, Supplementary interactive PCA plot). Laz speakers were within the range of variation of Georgian speakers, particularly those inhabiting eastern Georgia, which falls within the watershed of Mtkvari (Kura) River. Notably, Laz speakers currently inhabit the Black Sea coastal regions of NE Turkey and south-western Georgia. Svan speakers, which currently inhabit mountainous parts of north-western Georgia, were largely out of the range of Georgian-Laz variation. Megrelian speakers were mainly where Svan and Georgian-Laz clusters overlapped. Thus Kartvelian language speakers could be grouped into two major clusters: Georgian-Laz and Svan, with some overlap between these two. The Georgian-Laz group largely overlapped with modern Anatolians, while the Svan group was between modern North Caucasians and Iranians, but barely overlapped with them. Ancient South Caucasian genotypes dating from Neolithic, Copper, Bronze and Iron Ages were only available from the watersheds of Mtkvari and Araxes Rivers. The Georgian-Laz group was within the range of variation of these ancient South Caucasians. The Svan group kept almost the same distance from both these ancient South Caucasians and the Georgian-Laz group. The modern Kartvelian speakers were closer to Caucasus and Iranian hunter-gatherers than to hunter-gatherers from elsewhere. The Svan group was closer to the Caucasus hunter-gatherers than the Georgian-Laz group was. Bronze Age North Caucasians were far from the modern North Caucasians, and they rather clustered with the Neolithic, Bronze Age and modern South Caucasians from the watersheds of Mtkvari and Araxes Rivers.

## Discussion

Our best-fit phylogenetic model of Kartvelian languages, obtained through Bayesian phylogenetic inference, had a high posterior support and aligned with the widely accepted topology suggested by previous studies. However, in contrast to these studies, our model indicated earlier mean split dates in the evolutionary history of the languages. According to the mean split dates estimated by the phylogenetic model, the divergence between Svan and Karto-Zan occurred prior to or at the beginning of the introduction of metallurgy in the study area, while Georgian and Zan diverged in the Iron Age, specifically during the Urartian period. The mean age for the split of Zan into Megrelian and Laz was estimated at 1200 BP, which scholars attribute to the spread of Georgian and/or Georgian speakers from the watershed of Mtkvari (Kura) River to those of Chorokhi and Rioni Rivers^8,9^. The mean split time between Svan and Karto-Zan agrees with one of our assumptions that the split took place prior to the introduction of metallurgy, in an unidentified Kartvelian homeland. While we found considerable uncertainty in the inferred split dates of the best-fit Bayesian linguistic model, its average estimates are in better temporal agreement with past society and population genetic models than our other Bayesian linguistic models and those of previous studies are.

Co-occurrence of the wildlife elements, whose names are reconstructible to proto-Kartvelian names, largely coincided with the Upper Paleolithic refugia of woodlands and humans^72^. Assuming both the validity of our assumptions and Bayesian inference suggesting that the Svan and Karto-Zan split occurred around 8000 BP, the search for the Kartvelian homeland should be narrowed down to the Western Caucasus, Pontic Alps, Alborz, Zagros, Taurus, Mount Lebanon, Dinaric Alps, and Pindus. This is because the other areas of the taxa co-occurrence identified throughout the MaxEnt taxon-climate models appeared in the study area after 8000 BP.

The analyses of associations between the past human societies and biomes indicated that farming societies began their expansion in the study area around 11,000 BP, initiating a gradual displacement of hunter-gatherer communities. However, it was not until approximately 4000 BP that farming and metallurgical societies completely replaced hunter-gatherers. These technologically advanced societies exhibited a greater rate of expansion in regions with lower forest density, resulting in the displacement of hunter-gatherers towards more densely forested areas. Remarkably, only the middle-late Bronze Age and Iron Age societies were able to fully replace hunter-gatherers in their final stronghold located in the most densely forested areas, specifically those with a predominant presence of arboreal pollen exceeding 75%. The distribution of hunter-gatherers and farmers only overlapped in forested areas with less than 50% arboreal pollen until 8000 BP. However, from that point until 4000 BP (approximately 4000 years), there was minimal to no overlap between the two groups. Lifestyle differences and the prolonged absence of shared geographic areas between hunter-gatherers and farmers may have resulted in linguistic and genetic differences. Interestingly, these final strongholds of hunter-gatherer communities were located adjacent to the inferred areas of co-occurrence of the wildlife elements, whose names are reconstructible to proto-Kartvelian names (Fig. 3). Therefore, in search of the Kartvelian homeland, we also focused on these transboundary areas between hunter-gathering and farming communities.

The north–south and east–west gradients in the genetic variation of West Eurasian and Siberian populations across different periods implies some degree of geographic structure and regional continuity over time. Principal component analysis (PCA) of genome-wide SNP genotypes revealed two distinct linguo-genetic groups among the Kartvelian speakers, namely the Svan and the Georgian-Laz. The clusters of these groups were situated between the Caucasus hunter-gatherers on one end and the Neolithic-Copper Age Anatolians on the other. The Svan cluster exhibited a closer genetic affinity with the Caucasus hunter-gatherers, while the Georgian-Laz cluster displayed a stronger genetic resemblance to the ancient Anatolians. These observations are further corroborated by the studies of extant and Mesolithic/Epipalaeolithic populations^71^. Megrelian speakers seemed to be admixture between Svan and Georgian-Laz speakers. This genetic pattern aligns with the Bayesian linguistic tree such that the Georgian-Laz genetic group is the vector of the Karto-Zan linguistic branch and the Svan group is that of the Svan linguistic branch. Of all gene contributors, Caucasus hunter-gatherer-related ancestry has always been the major component of the South Caucasians over the last 8000 years—that is, since the Neolithic times^24,70^. Based on genetic studies, the arrival of Anatolian Neolithic farmers in the South Caucasus started ~ 8500 BP^73^. Subsequently, during the Copper Age, pastoralists from the Pontic-Caspian steppe arrived and established themselves in the Middle-to-Late Bronze Age period, laying the foundation for the emergence of the Armenian language^24^. The arrival of Anatolian farmers aligns more closely with our assumed timeframe for the divergence between Svan and Proto-Georgian-Zan and the assumed reasons behind this split related to the neolithization of the region. Following similar studies, we interpret our PCA outcome, assuming that all or most SNPs are neutral. On the contrary, the scrutiny of this assumption shows that many SNPs are functional or under selection (e.g.^74,75^). Nevertheless, even assuming that selection signatures are true and widespread, we are currently unaware of any theoretical models or practical demonstrations of positive or negative selection causing systematic shifts in genome-wide allele frequencies among human populations, of the type observed in our PCA figure. Instead, it is more likely that genetic drift and admixture, which operate on a genome-wide scale, offer a more plausible explanation for this pattern, which is also supported by archaeological evidence.

Here, we present a comprehensive scenario that effectively reconciles our findings, archaic lexical and toponymic data, as well as historical records. Prior to the neolithization of steppe and sparsely wooded areas in the Caucasus (i.e. prior to 8000 BP), which appears synchronous with the emergence of Neolithic Anatolia-related ancestry in the Caucasus, proto-Svan-Karto-Zan was spoken by hunter-gatherers around Rioni River—that is, an area between Mtkvari, Chorokhi and Enguri Rivers (Fig. 3). From 8000 BP until 4000 BP (approximately 4000 years) farming and metallurgical societies completely replaced hunter-gatherers in the Mtkvari watershed and most of the Chorokhi watershed, while hunter-gatherers remained in the Rioni and Enguri watersheds and some parts of the Chorokhi watershed, in what is now most of western Georgia (aka Colchis in the past). Archaeological evidence also confirms that during this period hunter-gatherers dominated in western Georgia, while farming and metallurgical societies thrived in eastern Georgia^76^ because these technologically advanced societies took a longer time to colonize dense forests typical of western Georgia (this study and^77^). Due to the 4000-year-long differences in lifestyle and environment, linguistic differences accumulated between societies inhabiting the Mtkvari and Chorokhi watersheds and those inhabiting the Rioni and Enguri watersheds. The language of the Rioni-Enguri group (i.e. hunter-gatherers) evolved into Svan, while that of the Mtkvari-Chorokhi group (i.e. farmers) evolved into Karto-Zan. This scenario explains why crop cultivation and sheep-breeding vocabulary differs between these groups. However, it fails to explicitly address the shared cattle-breeding vocabulary between the two groups. During prehistoric times, cattle inhabited not only lush grasslands but also forests, where they thrived near rivers and at forest edges, particularly in sedge beds^78^. In contrast, sheep dominated regions with drier conditions and less forest cover^79^. This suggests the possibility of some degree of cattle herding occurring in the Rioni-Enguri watersheds in the Neolithic, thus contributing to the shared vocabulary related to cattle breeding. Another explanation could be that the Kartvelian languages feature shared terms for cattle and red deer (*Cervus elaphus*), which might account for the commonality in cattle breeding terms. Thus, placing the Kartvelian homeland 8000 BP between Mtkvari, Chorokhi and Enguri Rivers supports our assumptions that part of the Kartvelian homeland initially was unsuitable for crop-cultivation and sheep-breeding, and the introduction of these practices in this part took long enough to cause and solidify the linguistic differences between this more forested part (i.e. the Rioni-Enguri watersheds) and the other less forested part (i.e. the Mtkvari-Chorokhi watersheds) of the Kartvelian homeland. Even today sheep-husbandry and the cultivation of predominant cereal crops (e.g. wheat, barley, rye, and oats) continue to be notably less prevalent in the Rioni-Enguri watersheds than in the Mtkvari-Chorokhi watersheds. This discernible disparity in farming practices is due to the persistently wetter climatic conditions characterizing the Rioni-Enguri watersheds.

As for the split of Karto-Zan, the speakers of this language most likely inhabited the watershed of Mtkvari (Kura) River, and that of Chorokhi (Çoruh) River before the Iron Age. Therefore, it is highly likely that Karto-Zan was spoken by pre-Kura-Araxes and Kura-Araxes farmers that thrived in the watershed of Mtkvari (Kura) River during the Copper and Bronze Ages. Over time, limited migration and communication between the Mtkvari and Chorokhi river watersheds caused the development of two distinct branches of Karto-Zan: Georgian in the Mtkvari watershed and Zan in the Chorokhi watershed. Although at present Georgian is spoken in most of the Chorokhi watershed, the pre-Georgian substrate in this region is Zan^9^. This divergence may have been a result of geographical barriers, such as the inclement mountain steppe and dense forests that separated these regions. Another factor that may have contributed to this communication impedance was the arrival of pastoralists, adapted to the cold steppe environment, from the Pontic-Caspian steppe. This coincided with the decline of the Kura-Araxes culture (aka Mtkvari-Araxes culture) and the emergence of the "Early Kurgan" culture^24,70^. These pastoralists likely entered the region through the western flank of the Caspian Sea, which provided a feasible corridor for ancient movements from the north of the Greater Caucasus ridge^71^. They subsequently settled in the mountain steppes of Armenia and north-eastern Turkey. Another plausible explanation for the lack of communication between these two groups is the rise of states such as the Iron Age kingdom of Urartu, the northern flank of which was situated between the Mtkvari and Chorokhi watersheds. Additionally, dense forests separated the watersheds from the north. Overall, the split of Karto-Zan into Georgian and Zan can be attributed to a combination of geographical barriers, the arrival of pastoralists from the Pontic-Caspian steppe, and the emergence of states like Urartu that linguistically was not Kartvelian. These factors contributed to the development of distinct linguistic and cultural branches within the region. Our scenario further suggests that Zan speakers from the Chorokhi watershed expanded to what now consists of Turkey’s Black Sea coastal regions and much of western Georgia. By the first century CE, their expansions gradually displaced Svan to the north, specifically into the upper reaches of Kodori, Enguri, Tskhenistskali, and Rioni Rivers. Subsequently Georgian and/or Georgian speakers spread from the Mtkvari watershed across the Pontic-Caspian divide in the seventh-eighth centuries CE, largely displacing Zan and splitting it into Laz and Megrelian, as well as further displacing Svan.

In summary, our estimation of mean split dates through Bayesian phylogenetic inference challenges the earlier conclusions drawn through lexicostatistical and glottochronological methods. Our analysis proposes that the split between these languages could have occurred earlier than it was thought before. We associate the divergence of Kartvelian languages with the interaction between landscape heterogeneity and important cultural and technological changes in the South Caucasus, such as the introduction of agriculture, metallurgy and state-run communities. Across the study area and the study time span Neolithic societies could not colonize dense forests, whereas Copper Age societies made limited progress in this regard, but not to the same degree of success achieved by Bronze and Iron Age societies.

The actual homeland of Indo-European languages has long been a mystery. Our findings may contribute significantly to narrowing down the search area for this homeland. Linguistic and population genetic studies point towards south of the Caucasus as the inferred location^4,8, 24,80^. Glacial refugia, where human populations sought shelter during the last glacial period, are believed to have significantly influenced the evolution and distribution of not only genetic but also linguistic diversity^72^. Glacial refugia appear to have a strong impact on linguistic family level differences prior to the Copper Age in our study area. Genetic and linguistic evidence suggests that the spread of Hattic and Hurrian languages are associated with ancient Anatolians and Levantines, respectively^24,81^. The geography of these ancient populations are strongly associated with the refugia, specifically the Anatolian and Levantine refugia^71^. The current study also suggests the importance of glacial refugia. Our analyses place the Kartvelian homeland in an area that intersects the Colchis glacial refugium in the South Caucasus. If refugia truly are sources of linguistic families and Indo-European languages originated somewhere south of the Caucasus, then the homeland of Indo-European languages can be refined to the Zagros or Hyrcanian (Alborz) refugia (Supplementary Fig. S4). These refugia are geographically closest to the South Caucasus^71,72^. The proposition of placing the Indo-European homeland in the Zagros and/or Hyrcanian refugia sheds light on the structural relationships or prolonged contacts between Kartvelian and Indo-European languages^4,8^.

Our study of genetic affinities using principal component analysis (PCA) indicates that Bronze Age North Caucasians were within the range of genetic variation of Bronze Age South Caucasians. Since then, North Caucasians appear to have shifted out of this range towards populations of Eurasian steppe and Siberia. This suggests substantial gene flow from Eurasian steppe and Siberia into the North Caucasus after the Bronze Age. Other studies also confirm this genetic shift due to post-bronze age admixture with populations from the Eurasian Steppe/Siberia^82^. This gene flow may explain some traces of Siberian languages in the North Caucasian, which led some linguists to propose that the North Caucasian, Yeniseian, Na-Dené and Sino-Tibetan languages are related^83,84^. Our multidisciplinary approach offers a unique opportunity to re-examine and enhance the existing genealogical models of intricate linguistic groups, such as Northwest and Northeast Caucasian languages.

### Supplementary Information

Supplementary Tables and Figures.

Supplementary Data 1.

Supplementary Data 2.

Supplementary Data 3.

Supplementary Data 4.

Supplementary Data 5.

Supplementary Data 6.

Supplementary Data 7.

Supplementary Data 8.

Supplementary Data 9.

Supplementary Data 10.

Supplementary Data 11.

Supplementary Data 12.

Supplementary Data 13.

Supplementary Data 14.

## Data Availability

All data generated or analyzed during this study are included in this article (and its Supplementary Information files). Any additional data related to this study are available from the corresponding author on request.

## References

[1] Pagel M, Atkinson QD, Calude AS, Meade A (2013). Ultraconserved words point to deep language ancestry across Eurasia. Proc Natl Acad Sci USA.

[2] Gamkrelidze, T., Machavariani, G. Sonat’ta sist’ema da ablaut’i kartvelur enebshi. saerto kartveluri st’rukt’uris t’ip’ologia. metsniereba, tbilisi [System of sonants and ablaut in the Kartvelian languages. Typology of the Common Kartvelian structure. Metsniereba, Tbilisi] (in Georgian), (1965).

[3] Klimov, G.A. O leksiko-statisticheskoy teorii M. Svodesha. V: Voprosy teorii yazyka v sovremennoy zarubezhnoy lingvistike. Moskva, Nauka: 239–253 [On the lexico-statistical theory of M. Swadesh. In: Questions of the theory of language in modern foreign linguistics. Moscow, Nauka: 239–253] (in Russian), (1961).

[4] Klimov, G.A. Etymological dictionary of the Kartvelian languages. Berlin; N.Y.: Mouton de Gruyter, (1998).

[5] Testelec, J.G. Sibilyanty ili kompleksy v prakartvel'skom? Voprosy yazykoznaniya 5: 10–28 [Sibilants or complexes in Proto-Kartvelian? Questions of linguistics 5: 10–28] (in Russian), (1995).

[6] Blažek V, Krpcová Š (2007). On the Application of Glottochronology to Kartvelian languages. Mother Tongue.

[7] Blažek V (2013). On classification of Kartvelian Languages. Folia orientalia.

[8] Gamkrelidze TV, Ivanov VV (1995). Indo-European and the Indo-Europeans: A Reconstruction and Historical Analysis of a Proto-Language and a Proto-Culture (Trends in Linguistics: Studies and Monographs [Tilsm]).

[9] Jorbenadze, B. Kartuli dialekt’ologia I. metsniereba, tbilisi [Georgian dialectology I. Metsniereba, Tbilisi] (in Georgian), (1989).

[10] Bouckaert R (2012). Mapping the origins and expansion of the Indo-European language family. Science.

[11] Campbell L, Poser WJ (2008). Language classification: History and method.

[12] Gray RD, Drummond AJ, Greenhill SJ (2009). Language phylogenies reveal expansion pulses and pauses in Pacific settlement. Science.

[13] Greenhill S (2015). An Online Database of New Guinea Languages. PLoS ONE.

[14] Hoffmann K, Bouckaert R, Greenhill SJ, Kühnert D (2021). Bayesian phylogenetic analysis of linguistic data using BEAST. Journal of Language Evolution.

[15] Klimov, G.A. Dva tysyacheletiya vneshney istorii malogo yazyka (svanskiye dannyye). Voprosy Yazykoznaniya 4: 19–24. nauka, moskva [Two Millennia of the External History of the Small Language (Svan Data). Questions of Linguistics 4: 19–24. Nauka, Moscow] (in Russian), (1996).

[16] Furnée EJ (1979). Vorgriechisch-Kartvelisches.

[17] Wichmann S, Müller A, Velupillai V (2010). Homelands of the world’s language families - A quantitative approach. Diachronica.

[18] Helmer, D., Gourichon, L., Monchot, H., Peters, J., Segui, M. S. In *The First Steps of Animal Domestication* (eds Vigne, J.-D. *et al.*) 86–95 (Oxbow Books, 2005).

[19] Verdugo MP (2019). Ancient cattle genomics, origins, and rapid turnover in the Fertile Crescent. Science.

[20] Yurtman E, Özer O, Yüncü E (2021). Archaeogenetic analysis of Neolithic sheep from Anatolia suggests a complex demographic history since domestication. Commun Biol.

[21] Baird D (2018). Agricultural origins on the Anatolian plateau. PNAS.

[22] Narasimhan, V. M. et al. The formation of human populations in South and Central Asia. Science 365, eaat7487. 10.1126/science.aat7487 (2019). 10.1126/science.aat7487 PMC6822619 31488661

[23] McGovern P (2017). Early Neolithic wine of Georgia in the South Caucasus. PNAS.

[24] Lazaridis I, Alpaslan-Roodenberg S (2022). The genetic history of the Southern Arc: A bridge between West Asia and Europe. Science.

[25] Chikobava, A. Zogadi enatmetsnierebis shesavali. tbilisis sakhelmts’ipo universit’et’is gamomtsemloba, tbilisi [Introduction to General Linguistics. TSU, Tbilisi] (in Georgian), (1952).

[26] Chumburidze, Z. Kartuli salit’erat’uro enis ist’oriis p’eriodizatsiisatvis. madli dedaenisa, tbilisi [For the periodization of the history of the Georgian literary language. Madli Dedaenisa, Tbilisi] (in Georgian), (1982).

[27] Gogolashvili, G., Arabuli, A. Akhali kartuli ena, ts’igni I. tbilisis sakhelmts’ipo universit’et’is gamomtsemloba, tbilisi [New Georgian language, book I. TSU, Tbilisi] (in Georgian), (2016).

[28] Kavtaradze, I. Kartuli enis ist’oriisatvis, XII-XVIII ss. tbilisis sakhelmts’ipo universit’et’is gamomtsemloba, tbilisi [For the history of the Georgian language, XII-XVIII centuries. TSU, Tbilisi] (in Georgian), (1964).

[29] Sarjveladze, Z. Kartuli salit’erat’uro enis ist’oriis shesavali. ganatleba, tbilisi [Introduction to the history of the Georgian literary language. Ganatleba, Tbilisi] (in Georgian), (1981).

[30] Shanidze, A. Subiekt’uri p’repiksi meore p’irisa da obiekt’uri p’repiksi mesame p’irisa kartul zmnashi. t’pilisis universit’et’is gamomtsemloba, t’pilisi [The subjective prefix of the second person and the objective prefix of the third person in the Georgian verb. TSU, Tbilisi] (in Georgian), (1920).

[31] Shanidze, A. dzveli kartuli enis gramat’ik’a. tbilisis universit’et’is gamomtsemloba, tbilisi [Grammar of the Old Georgian language. TSU, Tbilisi] (in Georgian), (1976).

[32] Haspelmath M, Tadmor U (2009). Loanwords in the World’s Languages: A Comparative Handbook.

[33] Heggarty, P. & Anderson, C. (eds) *Cognacy in Basic Lexicon (CoBL)*. (Max Planck Institute for the Science of Human History, Jena, 2019).

[34] Savelyev A, Robbeets M (2020). Bayesian phylolinguistics infers the internal structure and the time-depth of the Turkic language family. Journal of Language Evolution.

[35] Chukhua M (2019). Georgian-Circassian-Apkhazian Etymological Dictionary.

[36] Fähnrich, H. Sarjveladze, Z. Kartvelur enata et’imologiuri leksik’oni. s.s. "p’irveli st’amba", tbilisi [Etymological Dictionary of the Kartvelian Languages. Pirveli Stamba, Tbilisi] (in Georgian), (2000).

[37] Gamkrelidze, T. Sibilant’ta shesat’q’visobani da kartvelur enata udzvelesi st’rukt’uris zogi sak’itkhi. metsnierebata ak’ademiis gamomtsemloba, tbilisi [Sibilant correspondences and some problems of the oldest structure of the Kartvelian languages. Metsnierebata Akademiis Gamomtsemloba, Tbilisi] (in Georgian), (1959).

[38] Machavariani, G. Saerto kartveluri k’onsonant’uri sist’ema. tbilisis sakhelmts’ipo universit’et’is gamomtsemloba, tbilisi [The Common Kartvelian consonantal system. TSU, Tbilisi] (in Georgian), (1965).

[39] Melikishvili, I. kartvelur enata ori izolirebuli bgeratpardobis akhsnistvis. tanamedrove zogadi enatmetsnierebis sak’itkhebi 6: 70–86 [On the explanation of two isolated sound correspondences in the Kartvelian languages. Questions of modern general linguistics 6: 70–86] (in Georgian), (1981).

[40] Zhordania, R.G. Prinvelta svanuri t’erminologiisatvis. sakartvelos metsnierebata ak’ademiis moambe 47(2): 499–504 [For Svan terminology of birds. Bulletin of the Academy of Sciences of the Georgian SSR 47(2): 499–504] (in Georgian), (1967).

[41] Zhordania, R.G. Prinvelta megruli t’erminologiisatvis. sakartvelos sakhelmts’ipo muzeumis moambe 26–27A: 212–216 [For Megrelian Terminology of Birds. Bulletin of the State Museum of Georgia 26–27A: 212–216] (in Georgian), (1970).

[42] Abuladze, I. dzveli kartuli enis leksik’oni. metsniereba, tbilisi [Dictionary of Old Georgian Language. Metsniereba, Tbilisi] (in Georgian), (1973).

[43] Glonti, A. Kartul k’ilo-tkmata sit’q’vis k’ona. ganatleba, tbilisi [Dictionary of the Georgian dialects. Ganatleba, Tbilisi] (in Georgian), (1984).

[44] Kakhadze, O. p’ureulis leksik’a kartulshi. metsniereba, tbilisi [Vocabulary of cereals in Georgian. Metsniereba, Tbilisi] (in Georgian), (1987).

[45] Bouckaert R (2014). BEAST 2: a software platform for Bayesian evolutionary analysis. PLoS Comput. Biol..

[46] Alekseyenko AV, Lee CJ, Suchard MA (2008). Wagner and Dollo: a stochastic duet by composing two parsimonious solos. Syst. Biol..

[47] Chang W, Cathcart C, Hall D, Garrett A (2015). Ancestry-constrained phylogenetic analysis supports the Indo-European steppe hypothesis. Language.

[48] Hasegawa M, Kishino H, Yano T (1985). Dating of the human-ape splitting by a molecular clock of mitochondrial DNA. J. Mol. Evol..

[49] Tuffley C, Steel M (1998). Modeling the covarion hypothesis of nucleotide substitution. Math. Biosci..

[50] Heath TA, Huelsenbeck JP, Stadler T (2014). The fossilized birth-death process for coherent calibration of divergence-time estimates. Proc. Natl. Acad. Sci..

[51] Maturana, P. M., Russel, P. M., Brewer, B. J., Klaere, S. & Bouckaert, R. R. Model selection and parameter inference in phylogenetics using nested sampling. *Syst. Biol.* **68**, 219–233 (2019). 10.1093/sysbio/syy050 29961836

[52] Kass RE, Raftery AE (1995). Bayes factors. J Am Stat Assoc..

[53] Rambaut, A. et al. Tracer v. 1.6. Institute of Evolutionary Biology, University of Edinburgh, (2014).

[54] Rambaut, A. & Drummond, A. TreeAnnotator v. 2.3. 0. Part of the BEAST package, (2014).

[55] Holdridge LR (1947). Determination of world plant formations from simple climatic data. Science.

[56] Whittaker RH (1975). Communities and ecosystems.

[57] Karger, D.N., Conrad, O., Böhner, J., Kawohl, T., Kreft, H., Soria-Auza, R.W., Zimmermann, N.E., Linder, H.P., Kessler, M. Climatologies at high resolution for the earth’s land surface areas. Scientific Data 4(170122). 10.1038/sdata.2017.122, (2017). 10.1038/sdata.2017.122 PMC5584396 28872642

[58] Phillips SJ, Anderson RP, Schapire RE (2006). Maximum entropy modeling of species geographic distributions. Ecol Model.

[59] Elith J, Phillips SJ, Hastie T, Dudík M, Chee YE, Yates CJ (2011). A statistical explanation of MaxEnt for ecologists. Divers Distrib.

[60] Merow C, Smith MJ, Silander JA (2013). A practical guide to MaxEnt for modeling species' distributions: What it does, and why inputs and settings matter. Ecography.

[61] Elith J, Graham CH, Anderson RP, Dudik M, Ferrier S, Guisan A (2006). Novel methods improve prediction of species' distributions from occurrence data. Ecography.

[62] Phillips SJ, Dudik M (2008). Modeling of species distributions with Maxent: New extensions and a comprehensive evaluation. Ecography.

[63] Liu C, White M, Newell G (2013). Selecting thresholds for the prediction of species occurrence with presence-only data. Journal of Biogeography.

[64] Venables WN, Ripley BD (2002). Modern applied statistics with S.

[65] R Core Team. R. A language and environment for statistical computing. R Foundation for Statistical Computing, Vienna, Austria. http://www.R-project.org (2018).

[66] Breiman L (2001). Random Forests. Mach Learn.

[67] Wickham H (2016). ggplot2: Elegant Graphics for Data Analysis.

[68] Reich D, Price AL, Patterson N (2008). Principal component analysis of genetic data. Nature Genetics.

[69] Patterson N, Price AL, Reich D (2006). Population structure and eigenanalysis. PLoS Genet..

[70] Koptekin D (2023). Spatial and temporal heterogeneity in human mobility patterns in Holocene Southwest Asia and the East Mediterranean. Current Biology:.

[71] Gavashelishvili, A., Yanchukov, A., Tarkhnishvili, D. Murtskhvaladze, M., Akhvlediani, I. & Kazancı, C. Landscape genetics and the genetic legacy of Upper Paleolithic and Mesolithic hunter-gatherers in the modern Caucasus. Scientific Reports 11 (17985). 10.1038/s41598-021-97519-6 (2021). 10.1038/s41598-021-97519-6 PMC8429691 34504229

[72] Gavashelishvili A, Tarkhnishvili D (2016). Biomes and human distribution during the last ice age. Global Ecology and Biogeography.

[73] Skourtanioti E (2020). Genomic History of Neolithic to Bronze Age Anatolia, Northern Levant, and Southern Caucasus. Cell.

[74] Yuan D, Zhu Z, Tan X (2014). Scoring the collective effects of SNPs: association of minor alleles with complex traits in model organisms. Sci. China Life Sci..

[75] Huang S (2016). New thoughts on an old riddle: What determines genetic diversity within and between species?. Genomics.

[76] Meshveliani T (2013). On Neolithic origins in Western Georgia. Archaeology Ethnology & Anthropology of Eurasia.

[77] Kikvidze Z (2021). Traditional Ecological Knowledge in Georgia - A Short History of the Caucasus.

[78] van Vuure CT (2002). History, morphology and ecology of aurochs (*Bos taurus primigenius*). Lutra.

[79] Bendrey R (2011). Some like it hot: environmental determinism and the pastoral economies of the later prehistoric Eurasian steppe. Pastoralism.

[80] Heggarty, P. et al. Language trees with sampled ancestors support a hybrid model for the origin of Indo-European languages. Science 381 (6656). DOI: 10.1126/science.abg0818 (2023). 10.1126/science.abg0818 37499002

[81] Lazaridis I, Alpaslan-Roodenberg S (2022). A genetic probe into the ancient and medieval history of Southern Europe and West Asia. Science.

[82] Wang, C. et al. Ancient human genome-wide data from a 3000-year interval in the Caucasus corresponds with eco-geographic regions. Nat. Commun. 10.1038/s41467-018-08220-8 (2019). 10.1038/s41467-018-08220-8 PMC6360191 30713341

[83] Starostin, S.A. "On the Hypothesis of a Genetic Connection Between the Sino-Tibetan Languages and the Yeniseian and North Caucasian Languages''. In SHEVOROSHKIN, Vitaliy V. (ed.), Dene–Sino-Caucasian languages: materials from the First International Interdisciplinary Symposium on Language and Prehistory, Ann Harbor: Bochum: Brockmeyer, pp. 12–41 [Translation of Starostin 1984], (1991).

[84] Nikolaev, S.L. "Sino-Caucasian Languages in America''. In SHEVOROSHKIN, Vitaliy V. (ed.), Dene–Sino-Caucasian languages: materials from the First International Interdisciplinary Symposium on Language and Prehistory, Ann Harbor: Bochum: Brockmeyer, pp. 42–66 (1991).

