# Supplementary material for: The time and place of origin of South Caucasian languages: insights into past human societies, ecosystems and human population genetics
Source: Sci Rep. 2023 Nov 30;13:21133. doi: 10.1038/s41598-023-45500-w (PMC10689496; doi:10.1038/s41598-023-45500-w)

The time and place of origin of South Caucasian languages: Insights into past human societies, ecosystems and human population genetics

Alexander Gavashelishvili*, Center of Biodiversity Studies, Institute of Ecology, Ilia State University, Cholokashvili Str. 5, 0162 Tbilisi, Georgia
E-mail: aleksandre.gavashelishvili@iliauni.edu.ge

Merab Chukhua, Head of the Institute of Caucasiology, Faculty of Humanities, Ivane Javakhishvili Tbilisi State University, Ilia Chavchavadze Str. 1, 0162 Tbilisi, Georgia
E-mail: merab.chukhua@tsu.ge

Kakhi Sakhltkhutsishvili
Georgian DNA Project, Family Tree DNA
Ilia State University, Cholokashvili Str. 5, 0162 Tbilisi, Georgia
E-mail: Kajis2012@gmail.com

Dilek Koptekin
Department of Biological Sciences, Middle East Technical University, 06800 Ankara, Türkiye
E-mail: dilek.koptekin@metu.edu.tr

Mehmet Somel
Department of Biological Sciences, Middle East Technical University, 06800 Ankara, Türkiye
E-mail: msomel@metu.edu.tr

* Corresponding author: Alexander Gavashelishvili

Table S1. Confusion matrix of the climate-driven model of different biomes, estimated using the multinomial logistic regression (MLR).

		Predicted							
Observed		**Steppe**	**Desert**	**5-25% AP**	**25-50% AP**	**50-75% AP**	**>75% AP**	**Tundra**	**Glacier**
	**Steppe**	80	11	52	36	27	1	3	1
	**Desert**	12	23	1	0	0	0	0	0
	**5-25% AP**	10	0	238	6	0	0	0	0
	**25-50% AP**	16	0	18	142	1	0	0	0
	**50-75% AP**	4	0	0	16	113	1	0	0
	**>75% AP**	0	0	0	0	2	127	9	3
	**Tundra**	1	0	0	0	1	6	90	12
	**Glacier**	0	0	0	0	0	4	18	49

Figure S1. Boxplots of root ages (BP) across Bayesian phylogenetic linguistic models. Red diamonds plot mean values. Models are ordered such that their marginal likelihoods decrease from top to bottom. "*BinaryCTMC + StrictClock*" and "*BinaryCTMC + Gamma4 + StrictClock*" models are not displayed because they return unrealistically high root ages (> 100,000 BP).

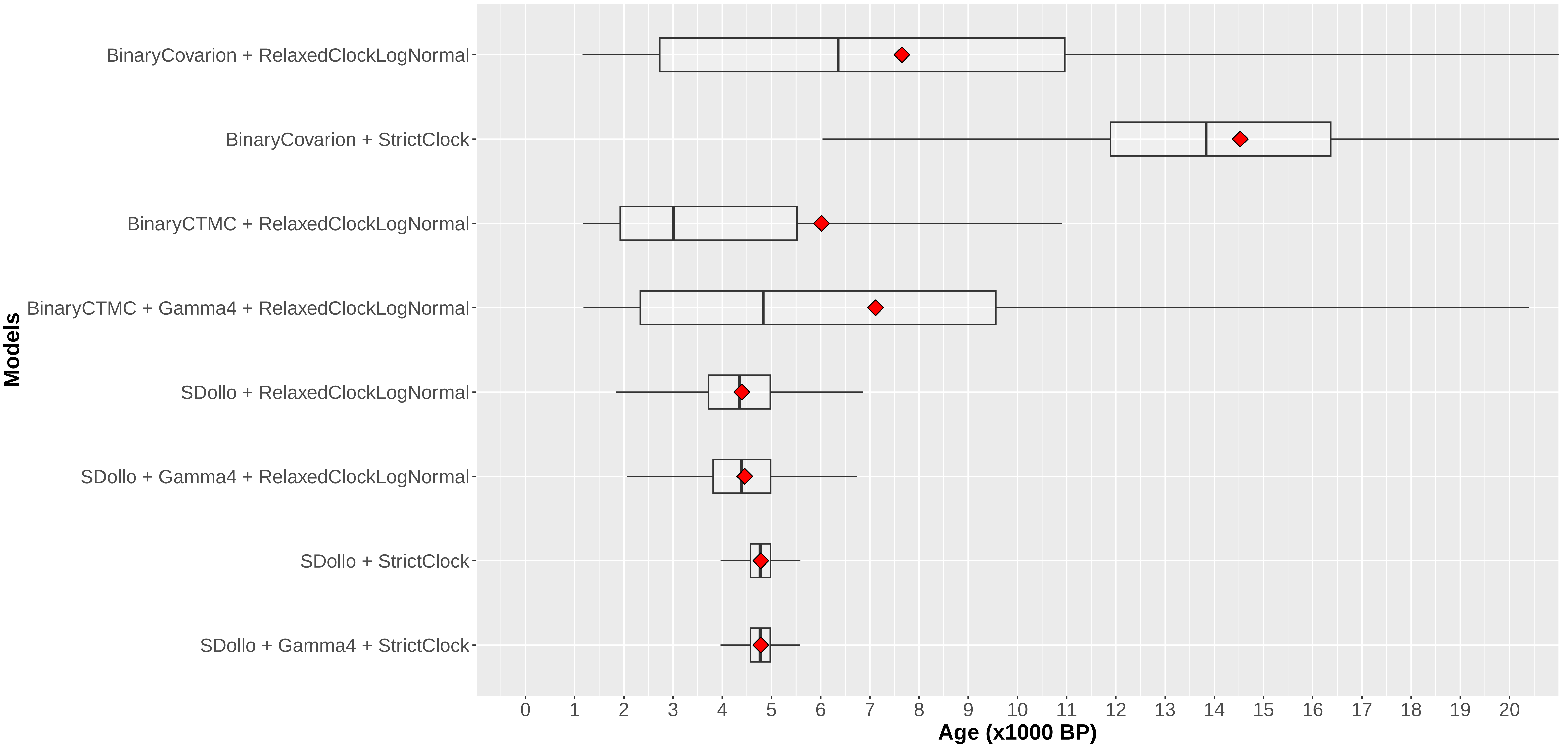

Figure S2. Co-occurrence of wildlife elements, whose names are reconstructible to proto-Kartvelian names, and the distribution of the past human societies in the Caucasus. The taxa co-occurrence is inferred by mapping each of the MaxEnt taxon-climate habitat suitability models, and subsequently binarizing and multiplying these maps. The co-occurrence areas were identified across CHELSA climatology time series, and then combined for each of the 5 time periods. The distribution of the past human societies is inferred from their associations with biomes (see Table 3 for details). The past societies are as follows: HG = Hunter-gatherers; N = Neolithic societies; CA = Copper Age societies; BA = Bronze Age societies; IA = Iron Age societies. The past human societies are mapped from biomes that are modeled across CHELSA climatology time series, and then aggregated for each of the 5 time periods using the mode value. The acronym of BP, denoting "Before Present", stands for years before 1 January 1950. The maps are generated using QGIS Desktop 3.22.7-Białowieża (https://qgis.org).

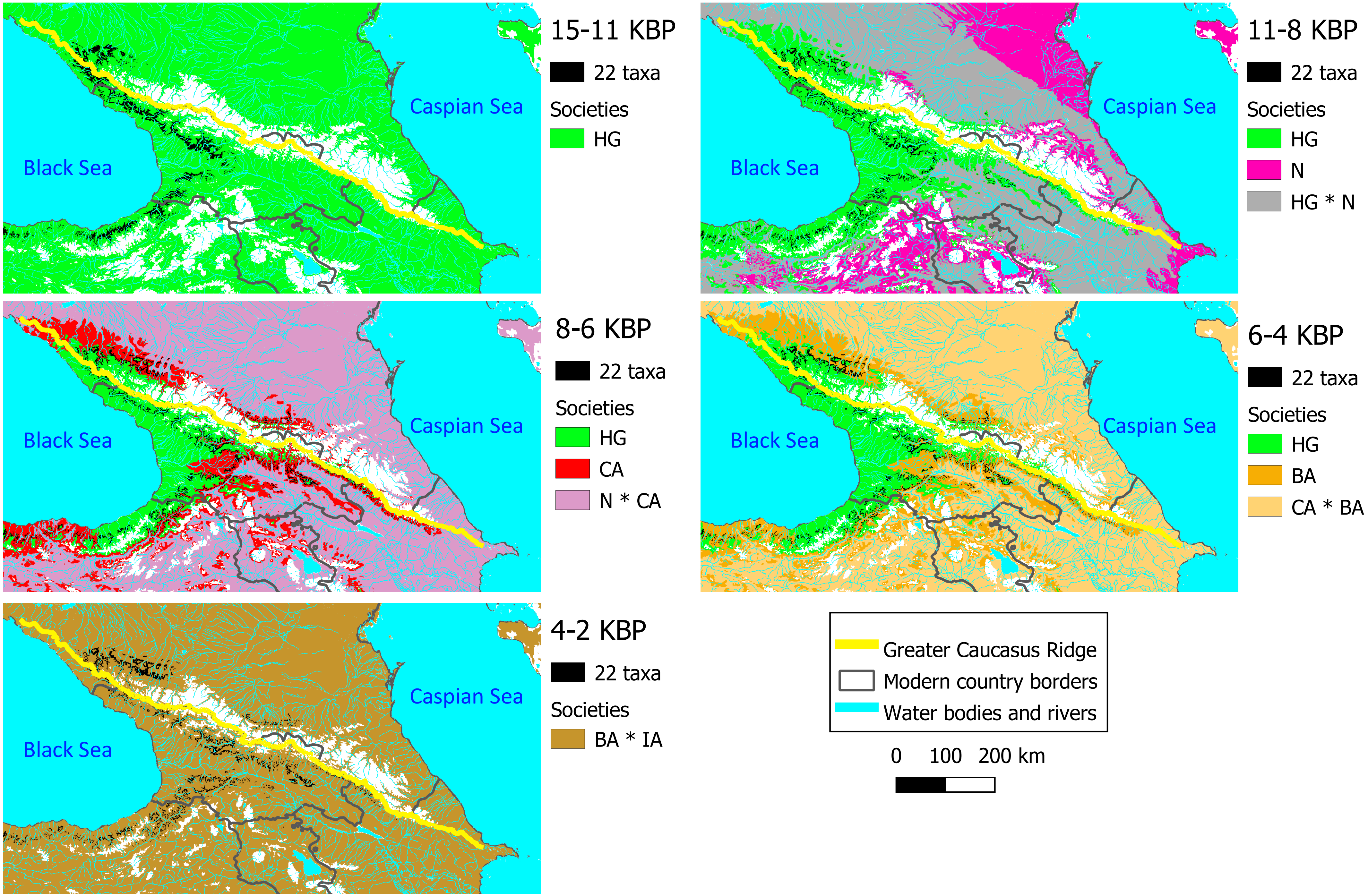

Figure S3. Principal component analysis (PCA) zooming in on speakers of modern Caucasian languages. The plot shows the first two principal components calculated using genomes of 969 individuals from modern West Eurasian populations (Koptekin et al. 2023), onto which a total of 478 ancient individuals from the Mesolithic/Epipalaeolithic period to the Iron Age (Koptekin et al. 2023) and 85 linguistically explicit modern individuals (Gavashelishvili et al. 2021) are projected. Georgian, Zan and/or Svan speakers with unknown linguistic ancestry or at least one ancestor from outside of their respective language-speaking population over the last five generations are assigned “Kartvelian”. HG = Hunter-gatherer; N = Neolithic; CA = Copper Age; BA = Bronze Age; IA = Iron Age; PR = Present-day) and speakers of 4 modern Kartvelian languages. PC1 correlates with the north-south differentiation, whereas PC2 correlates with the east-west differentiation across different periods.

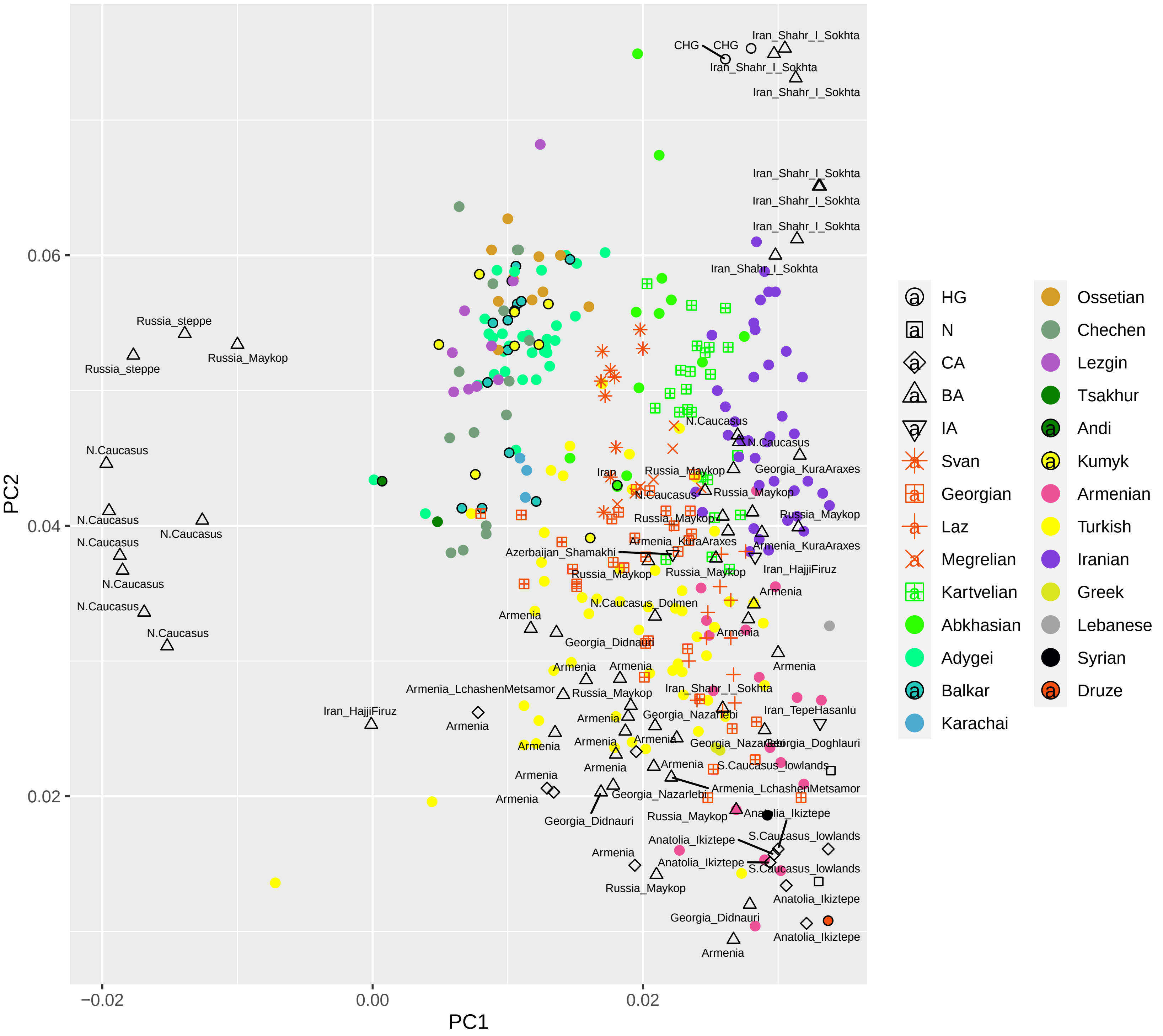

Figure S4. The refugia of savanna and dry woodland: these areas supported human source populations, while other areas acted as sinks in the source-sink metapopulation model of humans during the Last Glacial Maximum (extracted from Gavashelishvili and Tarkhnishvili 2016). The map is generated using QGIS Desktop 3.22.7-Białowieża (https://qgis.org).

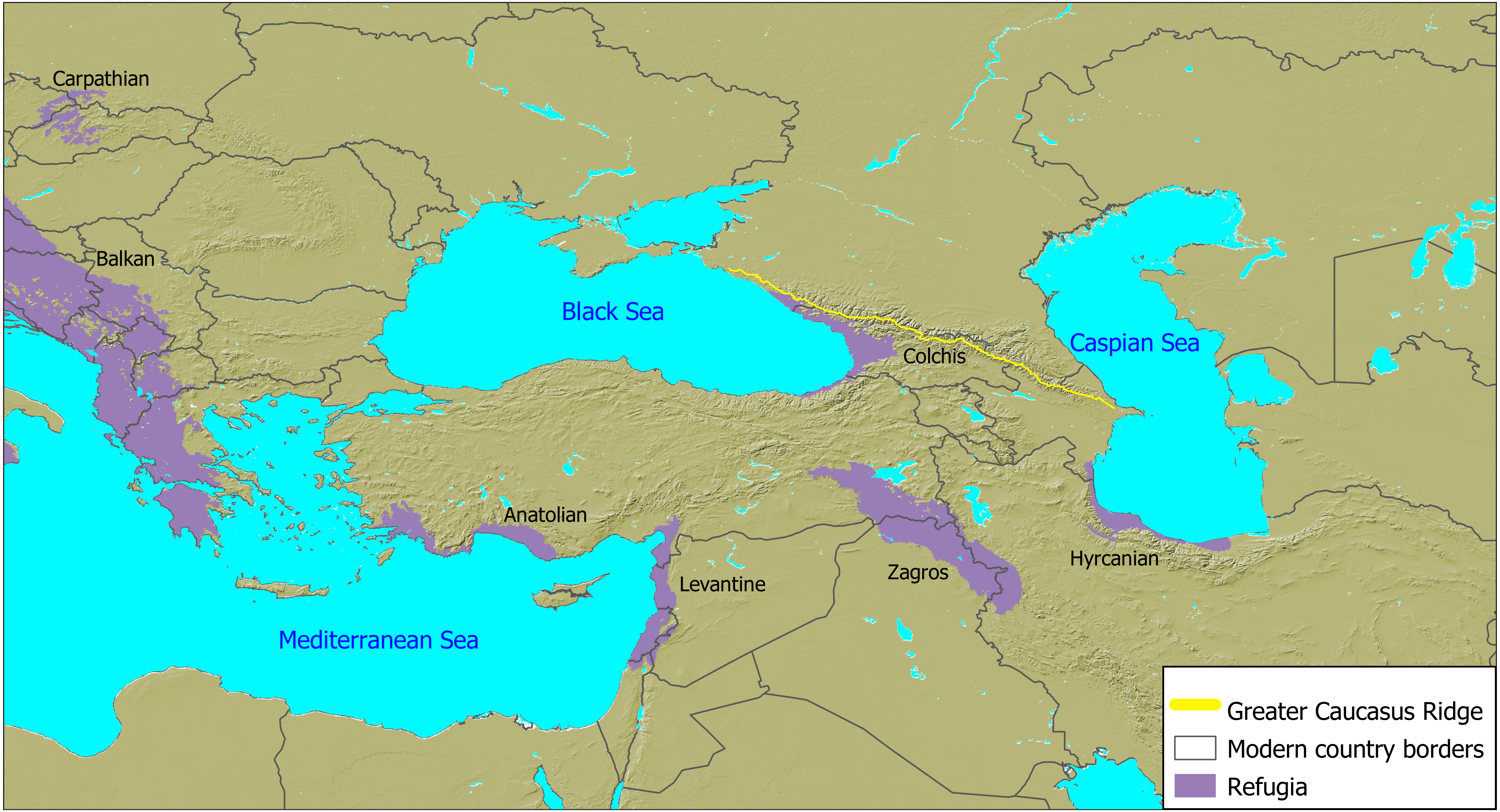

Supplement: Supplementary file 1 — Supplementary Tables and Figures. [file 41598_2023_45500_MOESM1_ESM.pdf]
