# Supplementary material for: The time and place of origin of South Caucasian languages: insights into past human societies, ecosystems and human population genetics
Source: Sci Rep. 2023 Nov 30;13:21133. doi: 10.1038/s41598-023-45500-w (PMC10689496; doi:10.1038/s41598-023-45500-w)
Supplement: Supplementary file 10 — Supplementary Data 9. [file 41598_2023_45500_MOESM10_ESM.docx]

**The time and place of origin of South Caucasian languages: Insights into past human societies, ecosystems and human population genetics**

Alexander Gavashelishvili*, Center of Biodiversity Studies, Institute of Ecology, Ilia State University, Cholokashvili Str. 5, 0162 Tbilisi, Georgia

E-mail: aleksandre.gavashelishvili@iliauni.edu.ge

Merab Chukhua, Head of the Institute of Caucasiology, Faculty of Humanities, Ivane Javakhishvili Tbilisi State University, Ilia Chavchavadze Str. 1, 0162 Tbilisi, Georgia

E-mail: merab.chukhua@tsu.ge

Kakhi Sakhltkhutsishvili

Georgian DNA Project, Family Tree DNA

Ilia State University, Cholokashvili Str. 5, 0162 Tbilisi, Georgia

E-mail: Kajis2012@gmail.com

Dilek Koptekin

Department of Biological Sciences, Middle East Technical University, 06800 Ankara, Türkiye

E-mail: dilek.koptekin@metu.edu.tr

Mehmet Somel

Department of Biological Sciences, Middle East Technical University, 06800 Ankara, Türkiye

E-mail: msomel@metu.edu.tr

* Corresponding author: Alexander Gavashelishvili

**Data files**

| **Data file** | **Explanation** |
| --- | --- |
| Supplementary Tables and Figures.pdf | This file contains **Table S1**, **Figure S1**, **Figure S2**, **Figure S3** and **Figure S4** |
| Supplementary Data 1.xlsx | Linguistic data to identify wildlife elements, whose current names can be traced back to proto-Kartvelian names |
| Supplementary Data 2.xlsx | Linguistic data to perform Bayesian phylogenetics to infer a dated phylogeny of the South Caucasian languages |
| Supplementary Data 3.csv | Data set of occurrence points for MaxEnt habitat suitability modeling |
| Supplementary Data 4.csv | Data set of background points for MaxEnt habitat suitability modeling |
| Supplementary Data 5.csv | Data on dated archaeological sites |
| Supplementary Data 6.csv | Data on dated sites of fossil pollen composition and land cover types |
| Supplementary Data 7.csv | Data on PCA of humans genotyped for genome-wide autosomal SNPs |
| Supplementary interactive PCA plot.html | Interactive plot of principal component analysis (PCA). The plot shows the first two principal components calculated using genomes of 969 individuals from modern West Eurasian populations (Koptekin et al. 2023), onto which a total of 478 ancient individuals from the Mesolithic/Epipalaeolithic period to the Iron Age (Koptekin et al. 2023) and 85 linguistically explicit modern individuals (Gavashelishvili et al. 2021) are projected. Data point colors differentiate geographic regions, while shapes identify archaeological periods. PC1 correlates with the north-south differentiation, whereas PC2 correlates with the east-west differentiation across different periods |
| Supplementary R codes for Data 5.txt | R codes to plot the distribution of the past human societies in relation to biomes and time across the study area |
| Supplementary R codes for Data 6.txt | R codes to perform Multinomial logistic regression (MLR) in order to evaluate the relationships between the biomized sites and the climatic predictors, and predict the MLR model to rasters of biomes across the study area and time span |
| Supplementary R codes for Data 7.txt | R codes to create PCA plots of humans genotyped for genome-wide autosomal SNPs |
| Kartvelian_alignment.nex | Input data extracted from **Supplementary Data 2.xlsx** for **BEAUti.exe** to create **Kartvelian_BEAST.xml** |
| Kartvelian_BEAST.xml | Input data for **BEAST.exe** to perform Bayesian phylogenetics in order to infer a dated phylogeny of the South Caucasian languages |

**Variables**

| **Variable** | **Explanation** |
| --- | --- |
| Taxon | Wildlife elements, whose current names can be traced back to proto-Kartvelian names |
| Latitude | Angular distance from the equator |
| Longitude | Angular distance from the Greenwich Meridian |
| bio01 | Mean annual air temperature (°C) |
| bio05 | Mean daily maximum air temperature of the warmest month (°C) |
| bio06 | Mean daily minimum air temperature of the coldest month (°C) |
| bio10 | Mean daily mean air temperatures of the warmest quarter (°C) |
| bio11 | Mean daily mean air temperatures of the coldest quarter (°C) |
| bio12 | Annual precipitation amount (kg m^-2^) |
| bio18 | Mean monthly precipitation amount of the warmest quarter (kg m^-2^) |
| bio19 | Mean monthly precipitation amount of the coldest quarter (kg m^-2^) |
| tri1km | Terrain Ruggedness Index (TRI), calculated from the SRTM 1-km elevation grid as the mean difference between a central pixel and its surrounding  cells using QGIS Desktop 3.22.7-Białowieża |
| Archaeological_Period | Archaeological periods, used in the analyses |
| Age_BP | Average years Before Present |
| biome | Biomes, used in the analyses |
| elevation | Elevation above sea level (m) |
| Region | Geographic regions |
| Population | Ethnic/sub-ethnic groups |
| PC1 | 1st principal component from PCA of humans genotyped for genome-wide autosomal SNPs |
| PC2 | 2nd principal component from PCA of humans genotyped for genome-wide autosomal SNPs |
